# Multi-Response Optimization of Aluminum Laser Spot Welding with Sinusoidal and Cosinusoidal Power Profiles Based on Taguchi–Grey Relational Analysis

**DOI:** 10.3390/ma18133044

**Published:** 2025-06-26

**Authors:** Saeid SaediArdahaei, Xuan-Tan Pham

**Affiliations:** Department of Mechanical Engineering, École de Technologie Supérieure, 1100, Notre-Dame Ouest Street, Montreal, QC H3C 1K3, Canada; saeid.saediardahaei.1@ens.etsmtl.ca

**Keywords:** keyhole, surface tension, darcy damping force, instability, optimization, RSD, GRA analysis, ANOVA

## Abstract

Laser weld quality remains a critical priority across nearly all industries. However, identifying optimal laser parameter sets continues to be highly challenging, often relying on costly, time-consuming trial-and-error experiments. This difficulty is largely attributed to the severe fluctuations and instabilities inherent in laser welding, particularly keyhole instabilities. This study examines the impact of laser power modulation parameters, which, when properly applied, have been found effective in controlling and minimizing process instabilities. The investigated parameters include different pulse shapes (sinusoidal and cosinusoidal) and their associated characteristics, namely frequency (100–800 Hz) and amplitude (1000–4000 W). The impact of these modulation parameters on keyhole mode laser spot welding performance in aluminum is investigated. Using a Taguchi experimental design, a series of tests were developed, focusing on eight key welding responses, including keyhole dimensions, mean temperature, and the variability of instability-inducing forces and related factors affecting process stability. Grey relational analysis (GRA) combined with analysis of variance (ANOVA) is applied to identify the optimal combinations of laser parameters. The results indicate that low amplitude (1000 W), low to intermediate frequencies (100–400 Hz), and cosinusoidal waveforms significantly enhance weld quality by improving process stability and balancing penetration depth. Among the factors, amplitude has the greatest impact, accounting for over 50% of the performance variation, followed by frequency and pulse shape. The findings provide clear guidance for optimizing laser welding parameters to achieve stable, high-quality aluminum welds.

## 1. Introduction

Aluminum and its alloys are widely used in industry due to their high strength-to-weight ratio, corrosion resistance, and excellent electrical conductivity [1]. Being nearly three times lighter than steel and about half the weight of titanium, they are particularly suitable for electric vehicle body structures and battery components [2].

Laser welding is widely adopted in the automotive and aerospace sectors for producing deep, precise welds in aluminum alloys [3,4]. However, challenges persist due to aluminum’s high reflectivity, thermal conductivity, and oxidation tendency [5]. Pulsed wave (PW) lasers help address these issues by using high initial energy density to overcome reflectivity while maintaining controlled average power to reduce overall heat input [6,7]. Keyhole-mode laser welding, a deep penetration technique for aluminum alloys, has been widely studied due to its complex process control. This mode typically initiates at a power density around 10^6^ W/cm^2^, where intense metal vaporization creates a keyhole and induces recoil pressure [8]. Keyhole-mode laser welding enhances productivity with welding speeds up to 20–30 times faster, while optimized parameters improve the mechanical properties of welded aluminum components [9].

Porosity is a common challenge in aluminum welding, mainly due to hydrogen solubility and keyhole instability. The evaporation of volatile alloying elements (e.g., Zn, Mg, and Li) further destabilizes the process [5]. Additionally, the narrow process window and rapid keyhole formation within microseconds complicate experimental studies due to erratic weld pool and keyhole behavior [10]. Inherent instabilities in keyhole laser welding limit its broader industrial adoption. Keyhole stability depends on the dynamic interplay of highly transient and fluctuating forces, such as surface tension (Marangoni and curvature effects), recoil pressure, hydrostatic and hydrodynamic pressures, and Darcy damping. Balancing these forces is essential to improve stability and reduce defects [11,12].

Laser power manipulation has shown effectiveness in controlling instabilities. Ardahaei et al. provided insights into the numerical modeling of keyhole instabilities in laser spot welding of aluminum using power wave modulation (PWM) [12,13]. Their study revealed that curvature effects and Darcy damping forces are key contributors to keyhole instability. Optimized pulse shapes, such as single- or quadruple-peak triangular and ramp-down rectangular pulses, effectively reduced spontaneous force fluctuations, enhancing process stability and minimizing defect probability. In a separate study, they demonstrated that rectangular pulses increased penetration depth by over 80% compared to continuous wave (CW) lasers, with power and frequency modulation significantly affecting keyhole behavior. Matasunawa et al. [14] showed that PWM considerably minimizes porosity by stabilizing keyhole and molten pool behavior. Tsukamoto et al. [15] further demonstrated that aligning modulation frequency with the molten pool’s natural oscillations significantly enhances keyhole stability and minimizes porosity formation. Zhang et al. [16] investigated sinusoidal power modulation in fiber laser welding of AZ31B magnesium alloy, reporting improved energy coupling, reduced underfill, refined microstructure, and enhanced mechanical properties due to improved keyhole and melt pool stability. Heider et al. [17] studied copper welding and found that sinusoidal power modulation, particularly at normalized frequencies of 0.2–0.4, reduced defects like pores and melt ejections by up to 90%, while improving keyhole stability. They optimized parameters such as modulation frequency, amplitude, and focal size to achieve deeper, more stable penetration.

A review of the literature indicates a gap in multi-response optimization for keyhole-mode laser welding of aluminum. While several researchers have proposed such techniques, most studies have lacked a comprehensive approach to simultaneously optimize multiple desired outcomes. Omoniyi et al. [18] applied the Taguchi method combined with grey relational analysis (GRA) to optimize the laser welding of Ti6Al4V, balancing bead geometry and micro-hardness. Their study highlighted GRA’s effectiveness in multi-objective welding problems where single-response optimization is insufficient. In another research, Tsai et al. [19] used Taguchi-GRA to optimize multiple quality attributes, such as roundness, taper, and HAZ, in the laser drilling of acrylic. By integrating diverse metrics into a single grey relational grade, the method effectively identified optimal parameters and balanced trade-offs among competing objectives, demonstrating GRA’s strength in multi-response optimization. This study presents a numerical investigation of keyhole-mode laser spot welding on aluminum, experimentally validated and optimized using Taguchi-based GRA. A novel cosinusoidal PWM profile was introduced and compared with continuous and sinusoidal profiles to assess its effectiveness in minimizing keyhole instabilities. GRA was employed to address the lack of robust multi-response optimization methods, enhancing simulation precision by reducing factors contributing to instability.

## 2. Experimental Details and Materials

**Figure 1:**
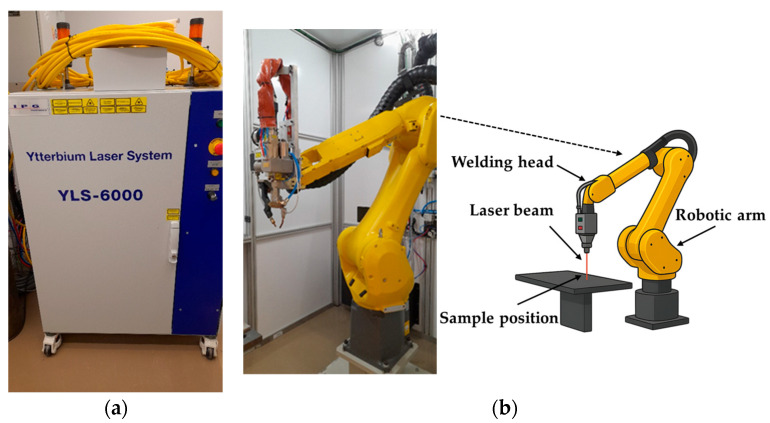
Experimental setup of the laser welding system: (**a**) laser source (IPG YLS-6000: Ytterbium Laser), (**b**) laser welding head and components.

Figure 1 shows the experimental setup for laser spot welding on aluminum using continuous, sinusoidal, and cosinusoidal pulse profiles. A fiber pulsed laser machine (IPG YLS-6000: Ytterbium Laser, IPG Photonics Corporation, Marlborough, MA, USA) with a maximum output laser power of 6 kW and a laser wavelength of 1090 nm was utilized to perform laser spot welds on test samples. The focal length of the laser was 300 mm, producing a beam diameter of 0.3 mm. The laser system used in this study was manufactured by IPG Photonics Corporation, headquartered in Marlborough, Massachusetts, United States. The material being used was Aluminum 6061 with a 2 mm thickness. A series of continuous, sinusoidal, and cosinusoidal laser pulses were used to create laser spots on the material. The surface was brushed prior to welding to remove contaminants and prevent oxidation. Due to aluminum’s high reflectivity, a 7.5 degree deviation was applied to the laser head to avoid damaging the welding head. The thermal properties of aluminum 6061 are listed in Table 1.

## 3. Methods and Configurations

**Figure 2:**
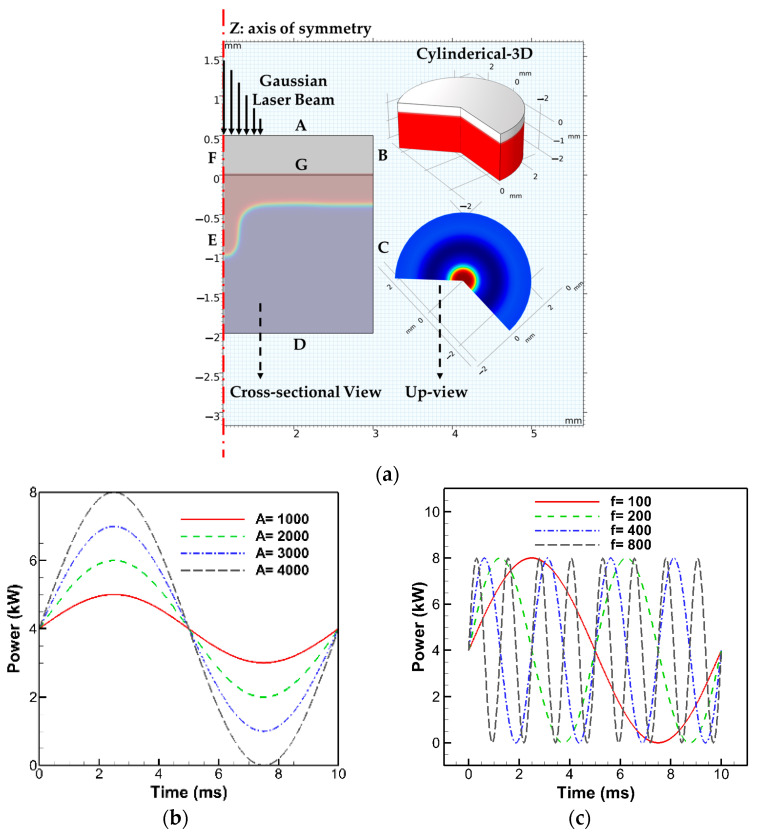

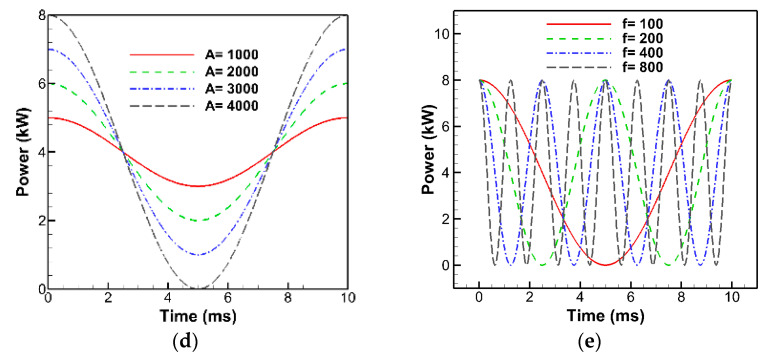
(**a**) Schematic 2D axisymmetric representation of numerical sample geometry and results in COMSOL Multiphysics. (**b**,**c**) Examples of sinusoidal power profiles under constant and variable amplitudes/frequencies. (**d**,**e**) Examples of cosinusoidal power profiles under constant and variable amplitudes/frequencies.

Laser spot welding was simulated using a 2D axisymmetric model in COMSOL Multiphysics 5.6, justified by the rotational symmetry of the stationary laser beam around the vertical *z*-axis. This setup allowed for efficient representation of 3D behavior via symmetry. This study investigated the pulse modulation impact by applying sinusoidal and cosinusoidal laser power distributions, focusing on keyhole stability and overall welding efficiency. The study applied sinusoidal and cosinusoidal laser power profiles to examine their impact on keyhole stability and welding efficiency. The computational domain and power profiles are shown in Figure 2. Boundaries ABGF and EGCD represent air and aluminum, respectively. A Gaussian beam was used to model the laser heat source.

### 3.1. Heat and Fluid Dynamics

A detailed description of the numerical methods, governing equations, and mesh sensitivity analysis used in this study is available and verified in our previous publications [12,13]. A brief summary is provided here, as the focus is on developing and applying a novel optimization technique. The modified level set (LS) method [22], modified mixture theory (MMT) [13,23], and the thermal enthalpy porosity technique (TEPT) [24,25] were used to model multiphase interactions during laser welding. These models were employed due to their proven effectiveness in dealing with phase transformations, keyhole morphology, and fluid flow. These approaches effectively capture vapor/liquid interactions (via LS/MMT) and solid/liquid interactions (via MMT/TEPT), ensuring reliable simulation results. MMT also facilitates finite element calculations across multi-phase elements applying mixture effects [13,23]. The model incorporated recoil pressure, surface tension forces, Darcy damping force, evaporation and mass loss, buoyancy, and surface dynamics. The numerical framework of this study was based on several assumptions to simplify the complex multiphase simulation: (a) Newtonian, incompressible laminar flow; (b) temperature-independent thermophysical properties; (c) mushy zone as a porous medium [12]; (d) neglecting multiple beam reflections; and (e) treating vaporized material as an ideal gas. The transport phenomena across all phases were modeled by solving the modified forms of energy (Equation (1)), mass (Equation (2)), and momentum (Equation (3)) conservation equations, and the LS (Equation (4)) equation.

(1) $$\rho C_{p}\frac{\partial T}{\partial t}+\rho C_{p}\vec{u}\cdot \nabla T=\nabla \cdot \left(k\nabla T\right)+(q_{Laser}-Q_{vapor})\delta \left(\phi \right)$$

(2) $$\nabla \cdot \vec{u}=\delta \left(\phi \right){\dot{m}}_{H-L}(\frac{\rho_{l}-\rho_{v}}{\rho^{2}})$$

(3) $$\rho \left(\frac{\partial \vec{u}}{\partial t}+\vec{u}\cdot \left(\vec{\nabla }\cdot \vec{u}\right)\right)=\vec{\nabla }\cdot [-pI+\mu (\vec{\nabla }\vec{u}+{\left(\vec{\nabla }\vec{u}\right)}^{T})])+\rho \vec{g}-\rho_{l}\beta_{l}(T-T_{melting})\vec{g}\phi -\mu_{l}K\vec{V}+(\gamma \cdot nk-\nabla_{s}\gamma \cdot t)\delta \left(\phi \right)$$

(4) $$\frac{\partial \phi }{\partial t}+\vec{u}\cdot \nabla \phi -\delta \left(\phi \right){\dot{m}}_{H-L}\left(\frac{V_{f,1}}{\rho_{v}}+\frac{V_{f,2}}{\rho_{l}}\right)+\gamma_{ls}\nabla \cdot (\phi \left(1-\phi \right)\frac{\nabla \phi }{\left|\nabla \phi \right|}-\epsilon_{ls}\nabla \phi )=0$$

The energy equation (Equation (1)) includes the velocity vector $\vec{u}$, specific heat capacity *C_p_*, temperature *T*, thermal conductivity *k*, and time *t*. The laser energy heat source *q_laser_* is defined as $(2\alpha P_{Laser}/\pi R_{eff}^{2})exp(-2r^{2}/R_{eff}^{2})B_{t}$ where $P_{Laser}$ is the peak laser power, $B_{t}$ is the analytic pulse-shaping function, α is the aluminum absorptivity, and $R_{eff}$ is the effective beam radius. Energy loss due to evaporation, *Q_vapor_*, is calculated as $-L_{V}{\dot{m}}_{H-L}$, where $L_{V}$ is the latent heat of vaporization, and ${\dot{m}}_{H-L}$ refers to the mass loss due to evaporation, defined as $\left(1-\beta_{r}\right)\;\sqrt{M/2\pi R}(P_{sat}\left(T\right)/\sqrt{T})$. The saturated vapor pressure $P_{sat}$ is determined by $P_{atm}\exp\left[\left(1-(T_{v}/T)\right)ML_{v}/RT_{v}\right]$ [26]. Here, $\beta_{r}$ is the retro-diffusion coefficient, *R* the universal gas constant, and *M* the molar mass of vaporized particles. A delta function $\delta \left(\phi \right)$, characterized using the LS variable ϕ, applies the laser heat flux and energy loss due to evaporation only at the vapor/liquid interface (where ϕ=0.5). Equation (2) represents the modified mass conservation equation incorporating a source term to add the impact of recoil pressure [25,27]. Equation (3) represents the momentum conservation, including the dynamic viscosity μ, pressure and viscous stresses, gravity, buoyancy, Darcy damping, and surface tension forces. The Darcy drag coefficient K, is defined with a relation as $((180/d^{2}){\left(1-V_{l}\right)}^{2})/{{(V}_{l}}^{3}+b$)), with *d*, the dendrite size constant set to 10^−2^ cm [28], and *b*, the constant used to avoid division by zero. $\beta_{l}$ and ${\left(\vec{\nabla }\vec{u}\right)}^{T}$ are the volume thermal expansion coefficient and the transpose of velocity vector gradient. $V_{l}$ is the liquid volume fraction, assigned to one above the liquidus temperature, zero below the solidus temperature, and $\left(T-T_{s}\right)/\left(T_{l}-T_{s}\right)$ between the two [24,29,30]. Equation (4) is the modified LS equation, improved by adding a gas dynamic source term to model vaporization effects due to mass loss and vapor pressure at the vapor/liquid interface [22]. Parameters $\gamma_{ls}$ and $\epsilon_{ls}$ are the reinitialization and the interface thickness values, respectively, set based on sensitivity analysis [13]. $V_{f,1}$ and $V_{f,2}$ denote the gas and solid/liquid volume fractions.

### 3.2. Optimization Approach

The Taguchi-based GRA was performed to optimize the numerical data from cosinusoidal and sinusoidal power profiles for enhancing keyhole laser welding performance on aluminum.

#### 3.2.1. Taguchi Design

The Taguchi method enhances product quality through system, parameter, and tolerance design strategies. This study adopted parameter design, focusing on identifying robust parameter settings through controlled experimentation. An orthogonal array (OA) was used to structure the experimental layout, minimizing the number of simulations. The selection of OA was guided by computational cost, time constraints, and study objectives [31]. The total degrees of freedom (DOF) of the experiment are defined in Equation (5).

(5) $${DOF}_{experiment}=\sum {DOF}_{factor}+\sum {DOF}_{interactions}$$

Table 2 lists the factors and levels used in the Taguchi design. The interaction DOF between two factors was calculated as the product of their individual DOFs, where each factor’s individual DOF was one unit less than its number of levels.

This study considered three factor interactions: A×B, A×C, and B×C. The total DOF yielded ${\left[\left(2-1)+2\times (4-1\right)\right]}_{{DOF}_{factor}}+{\left[2\times \left(1\times 3\right)+(3\times 3)\right]}_{\sum {DOF}_{interactions}}=22$. A suitable OA must have a DOF equal to or greater than this value. Accordingly, an $L_{32}$ OA was selected to fully capture all main and interaction effects influencing the keyhole welding process, as shown in Table 3.

#### 3.2.2. Grey Relational Analysis (GRA)

The primary objective of this study was to optimize the welding process by minimizing keyhole instability. Our previous work confirmed that large fluctuations in surface tension, Darcy damping force, and velocity significantly increase the likelihood of keyhole collapse by promoting instability [12]. Additionally, parameters such as mean temperature, keyhole depth, and width are critical for ensuring mechanical integrity and welding efficiency. Therefore, a multi-response optimization technique was developed to simultaneously account for these key parameters and their desired behaviors.

- Grey relational analysis (GRA)

The GRA approach is a technique that takes into account all the responses and combines them into one particular response by transforming them into Grey relational grades (GRGs) [18,19]. This study aimed to optimize desirable responses, such as keyhole depth, width, and mean temperature, while minimizing relative standard deviations (RSDs) of the Darcy damping force, surface tension, and velocity as key contributors to instability. The response objectives are summarized in Table 4. Preprocessing involved converting the raw data into a comparable format. The equations for calculating the standard deviation (SD) and RSD of the fluctuating forces and velocity are shown in Equations (6) and (7). The GRG computation followed three steps: normalization, Grey relational coefficient (GRC) calculation, and final GRG evaluation, with normalization performed using either Equation (8) or Equation (9), depending on the objective type being chosen as ‘larger-the-better’ or ‘smaller-the-better’ [32].

(6) $${SD}_{j}^{\left(i\right)}=\sqrt{1/n\sum_{t=1}^{n}{\left(x_{j}^{\left(i\right)}\left(t\right)-{\bar{x}}_{j}^{\left(i\right)}\right)}^{2}}$$

(7) $${RSD}_{j}^{\left(i\right)}=({SD}_{j}^{\left(i\right)}/{\bar{x}}_{j}^{\left(i\right)})\times 100$$

(8) $${X_{ij}}^{*}=\left(X_{ij}-\min(X_{j}\right)/\left(\max X_{j}-\min X_{j}\right)$$

(9) $${X_{ij}}^{*}=\left(\max X_{j}-X_{ij}\right)/\left(\max X_{j}-\min X_{j}\right)$$

In Equations (6) and (7), $x_{j}^{\left(i\right)}\left(t\right)$ denotes the time-dependent value of the *j*-th response (e.g., surface tension, Darcy damping force) for the *i*-th test at time step *t*, and ${\bar{x}}_{j}^{\left(i\right)}$ represents its mean over all *n* = 1000 simulation steps sampled from *t* = 0 to *t* = 0.01 s. In Equations (8) and (9), $X_{ij}$ and ${X_{ij}}^{*}$ are the original and normalized values of the *j*-th response of the *i*-th test. $X_{j}$ is defined as a set of all values of the *j*-th response across all 32 experiments or test cases ($X_{j}=\left\{X_{1j},X_{2j},\ldots X_{32j}\right\}$). All the response values were scaled into [0, 1] using these equations (Equations (8) and (9)).

The next steps are devoted to calculating the deviation from the ideal normalized value and the GRC, which are shown in Equations (10) and (11) [32]. Finally, the GRG can be calculated using Equation (12).

(10) $${∆}_{ij}=\left|{X_{ij}}^{*,ideal}-{X_{ij}}^{*}\right|$$

(11) $${GRC}_{ij}={(∆}_{min}+{\xi ∆}_{max})/\left({∆}_{ij}+{\xi ∆}_{max}\right)$$

(12) $${GRG}_{i}=(1/m)\sum_{j=1}^{m}{GRC}_{ij}$$

where ${∆}_{ij}$ denotes the deviation of the normalized response from the ideal normalized response (typically taken as 1), while ${∆}_{min}$ and ${∆}_{max}$ represent the minimum and maximum possible deviations, usually set to 0 and 1. The distinguishing coefficient ξ, ranging from 0 to 1, was set to 0.5 for stability [18,32]. In Equation (12), *m* is the total number of responses considered in this analysis (here, *m* = 8).

- Analysis of variance (ANOVA)

The performance of the tests, factors, and levels in the Taguchi method was analyzed using a statistical measurement approach known as ANOVA. This quantified the contribution of each factor, their main effects, interactions, and associated errors on the overall response. Since the optimization data were obtained from single-run numerical simulations, standard ANOVA was applied without considering noise factors. The analysis assessed the statistical significance of each factor on the optimized response, represented by the grey relational grade (GRG).

### 3.3. System, Software, and Calculation Details

COMSOL Multiphysics 5.6 was used for simulations on a Lenovo ThinkStation P720 equipped with an Intel^®^ Xeon^®^ Gold 5118 CPU (12 cores, 24 threads) and 128 GB RAM. The CPU was produced by Intel corporation, based in Santa Clara, California, USA. The system was assembled by LENOVO, headquartered in Beijing, China. Three physics interfaces, including Heat Transfer in Fluids, Laminar Flow, and Level Set, were employed and coupled using the Non-Isothermal Flow and Two-Phase Flow interfaces. These ensured accurate modeling of three-phase phenomena by linking the heat transfer, fluid flow, and level set dynamics. A time step of 10 μs was used, with an extra-fine mapped mesh (0.02 mm quadrilateral elements) optimized for fluid dynamics. PARDISO solvers were applied: a nested dissection multithreaded version for fluid flow and an automatic preordering version for the Heat Transfer and Level Set equations. The optimal interface thickness and reinitialization parameter for the level set method were set to 5 m/s and 0.03 mm, respectively, ensuring better computational efficiency. All mathematical calculations related to the optimization technique were performed using Microsoft Excel.

## 4. Results and Discussion

This section presents the experimental and numerical results, optimization outcomes, and ANOVA analysis for laser spot welding on aluminum using sinusoidal and cosinusoidal power profiles. Eight key criteria were used to guide the optimization, with emphasis on minimizing keyhole instabilities and enhancing penetration depth and width. Parameters such as the mean temperature, RSDs of surface tension and Darcy damping forces, maximum fluid velocity, and keyhole geometry were analyzed to determine the optimal welding conditions.

### 4.1. Experimental Results and Validation

**Figure 3:**
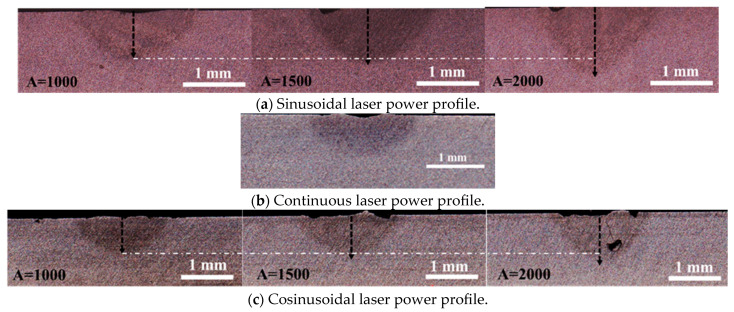
Cross-sectional view of the samples under different laser power profiles using an identical total laser energy of 40 j (4 kW over 10 ms) for (**a**) sinusoidal laser power profile under various laser power amplitudes, (**b**) continuous laser power profile, and (**c**) cosinusoidal laser power profile under various laser power amplitudes.

**Figure 4:**
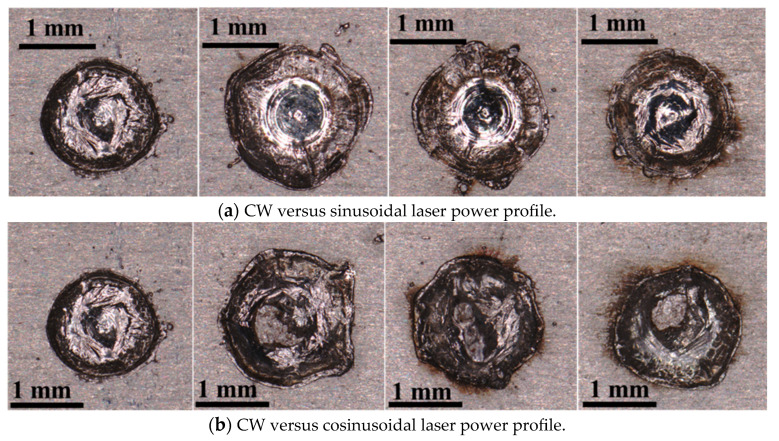
Top view of the laser spots produced under CW and sin/cosinusoidal laser power profiles for (**a**) comparison of CW with sinusoidal laser power profiles with varying laser power amplitudes (left to right: CW, Sin: A = 2000, 1500, 1000) and (**b**) comparison of CW with cosinusoidal laser power profiles with varying laser power amplitudes (left to right: CW, Cos: A = 2000, 1500, 1000).

The effects of the sinusoidal and cosinusoidal laser power profiles were evaluated against the conventional continuous profile through laser spot welding trials. Cross-sectional and top-view images (Figure 3 and Figure 4) illustrate that both the weld depth and width increased with modulated profiles. Specifically, the depth and width increased from 0.755 mm and 1.788 mm (continuous) to 0.900 mm and 1.985 mm (cosinusoidal, 2000 W amplitude), and further to 1.119 mm and 2.011 mm (sinusoidal, 2000 W amplitude). Moreover, increasing the amplitude from 1000 W to 2000 W at 100 Hz further enhanced the weld geometry. For the cosinusoidal profiles, the depth and width increased from 0.781 mm and 1.874 mm to 0.900 mm and 1.985 mm; for the sinusoidal profiles, they increased from 0.813 mm and 1.890 mm to 1.119 mm and 2.011 mm.

Figure 4 presents top-view images of the laser spots under different power profiles. The weld width increased with both sinusoidal and cosinusoidal profiles and further widened with higher power amplitudes compared to the continuous mode. These results confirm the superiority of modulated profiles in achieving greater depth-to-width ratios using the same total laser energy.

**Figure 5:**
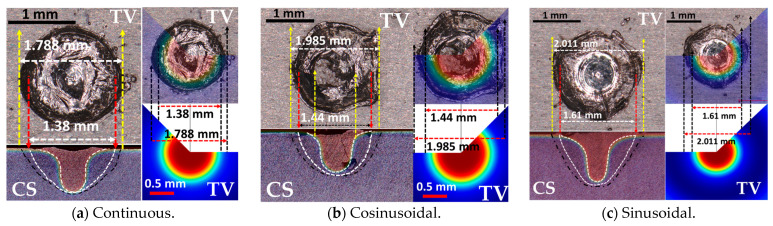
The numerical and experimental results of the top view and cross-section comparisons for (**a**) CW, (**b**) cosinusoidal (A = 2000, f = 100), and (**c**) sinusoidal (A = 2000, f = 100); the black dashed lines in the cross-section images represent the weld zone and HAZ, while the white dashed lines represent the numerically-derived weld zone and keyhole lines. CS is short for cross-section view, and TV is the top view.

The numerical simulation was validated by comparing the simulated weld width and depth with the experimental results for three cases: the sinusoidal, cosinusoidal (100 Hz, 2000 W), and continuous power profiles. As shown in Figure 5, the simulated keyhole and molten pool dimensions closely matched the experimental measurements, with minimal error. This strong agreement confirms the reliability of the simulation approach in accurately predicting weld geometry and keyhole behavior.

### 4.2. Numerical Results and Optimization Procedures

**Figure 6:**
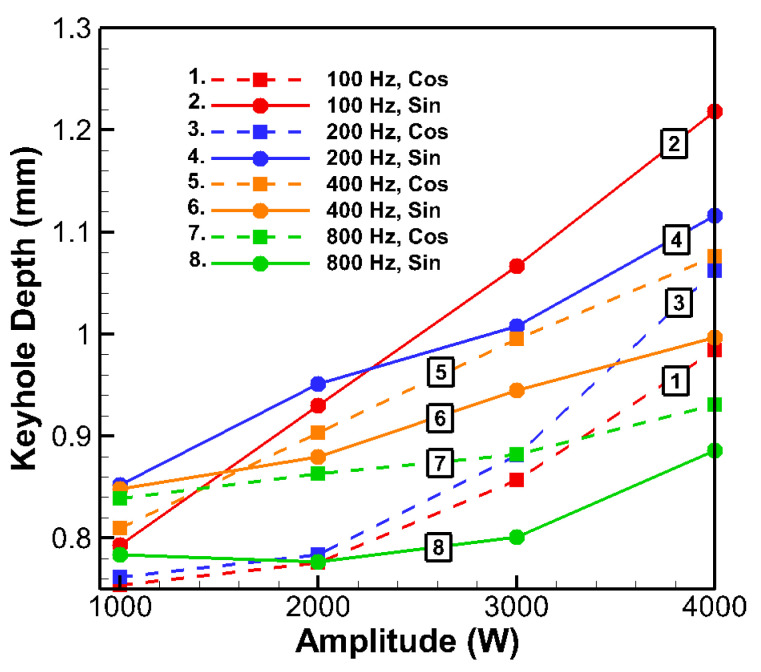
Keyhole depth versus amplitude at different frequencies for sinusoidal and cosinusoidal laser profiles.

Following the confirmed improvements in weld geometry using the sinusoidal and cosinusoidal profiles, the numerical study was extended to explore various frequencies and amplitudes under a constant total laser energy of 40 J for consistency. Using the Taguchi method (Table 2 and Table 3), 32 simulations were performed. GRA and ANOVA were applied to determine the optimal welding parameters. The numerical results for the eight optimization criteria are summarized in Table 5 and Figure 6, with GRA-related and ANOVA data presented in Table 6 and Table 7.

#### 4.2.1. Keyhole Depth and Width

Analysis of Table 5 shows that the keyhole depth was primarily influenced by the laser power amplitude, with pulse frequency and shape having secondary effects. Increasing the amplitude consistently deepened the keyhole across all profiles. For example, at 100 Hz with sinusoidal pulses, raising the amplitude from 1000 W to 4000 W increased the depth by 54% (0.793 mm to 1.218 mm). A similar trend occurred for other frequencies (Figure 6), though the magnitude of the depth increase diminished at higher frequencies (e.g., 800 Hz). Physically, at lower frequencies, longer pulse durations allowed high-amplitude peaks to deliver sustained energy, resulting in deeper keyholes. In contrast, at 800 Hz, where each pulse lasted approximately 1.25 ms, the shorter duration limited the energy delivered per pulse, reducing the effectiveness of higher peak power on increasing depth. Frequency alone had a mild impact on the depth at low amplitudes but showed a stronger effect at higher amplitudes. For instance, sinusoidal depth dropped by 27% with increasing frequency from 100 Hz to 800 Hz at 4000 W. Cosinusoidal depth also declined but less significantly (5%). This suggests a strong interaction between frequency and amplitude, which is important for optimization. Overall, frequencies from 100 to 400 Hz yielded comparable depths (around 0.9–1.0 mm at mid-to-high amplitudes), with a notable drop at 800 Hz. The slight decline in depth with very high frequency can be attributed to reduced energy per pulse and rapid cycling, which prevented the keyhole from fully developing before the pulse ended. The pulse shape had a subtle yet frequency-dependent influence on penetration. On average, sinusoidal pulses yielded slightly deeper welds (0.928 mm vs. 0.885 mm). At 100 Hz and 4000 W, the sinusoidal profiles produced 24% deeper penetration than the cosinusoidal profiles (1.218 mm vs. 0.984 mm), likely due to their gradual power increase, which facilitated stable keyhole formation before delivering the peak energy, enhancing penetration. Conversely, at 800 Hz, cosinusoidal pulses outperformed the sinusoidal pulses (0.931 mm vs. 0.886 mm), as early power peaks became more effective during brief pulse durations. Overall, frequencies between 100 and 400 Hz at mid-to-high amplitudes provided the most consistent depth performance.

The keyhole width followed trends similar to the depth but showed smaller relative variations. Higher amplitudes slightly increased the width; for example, at 100 Hz, sinusoidal pulses widened the keyhole from 1.50 mm to 1.73 mm (15% increase), compared to a 54% increase in depth. The width generally decreased with higher frequencies. Sinusoidal pulses at 100 Hz produced the widest melt pools (≈1.5 mm at low amplitude), while 800 Hz pulses yielded narrower widths (≈1.35 mm). This trend reflects the effects of longer, more energetic pulses promoting lateral heat diffusion, whereas high-frequency pulsing concentrated energy locally.

#### 4.2.2. Thermal Response (Mean Temperature)

The mean material temperature showed a clear dependence on the pulse parameters. An increasing amplitude consistently reduced the mean temperature across all frequencies. For example, with sinusoidal pulses at 100 Hz, the mean temperature dropped from 2682 K at an amplitude of 1000 W to 1941 K at an amplitude of 4000 W. Similar reductions (≈500–600 K) were observed for cosinusoidal pulses and at other frequencies (Table 5). This inverse relationship is attributed to higher amplitude pulses causing intense but brief heating, followed by longer cooling periods, which lowers the time-averaged temperature. Thus, higher amplitudes not only enhanced penetration but also promoted lower mean temperatures, which may benefit heat-sensitive materials. Conversely, higher frequencies increased the mean temperature by reducing the time between pulses and promoting more continuous energy input. The pulse shape had a negligible direct impact on the mean temperature, which was expected since shape altered the temporal distribution within a pulse but not the overall energy per pulse in these tests.

#### 4.2.3. Process Stability and Fluctuations (Velocity and Forces)

Table 5 reveals that the pulse parameters significantly influenced process stability. Amplitude was the primary contributor to instability, with increased values consistently raising the relative standard deviation (RSD) of key forces. For instance, increasing the amplitude from 1000 W to 4000 W more than doubled the RSD of surface tension force on average (from ~30% to ~57%). Physically, a larger amplitude means higher peak power, causing stronger vapor recoil pressure and fluid flow surges during the pulse peak, followed by a low-power period producing a violent stirring of the melt pool, leading to greater variability. Pulse frequency had a dual effect. Higher frequencies generally reduced the RSDs of surface tension and Darcy damping forces (e.g., averaging over shapes, radial surface tension RSD dropped from 52.7% at 100 Hz to 40.3% at 800 Hz), likely due to more frequent but smaller perturbations. However, the RSD of fluid velocity increased with frequency, from ~41% at 100 Hz to ~58% at 800 Hz (averaging over shapes) as rapid pulsing kept the molten pool in continuous motion, preventing flow stabilization. These frequency effects highlight that different aspects of the process responded differently to the pulse rate: the instantaneous flow became more vigorous with fast repetition, yet the forces driving or resisting that flow might have fluctuated less extremely due to the more continuous application. Pulse shape also played a role in stability, especially at low frequencies. At 100 Hz and 4000 W, cosinusoidal pulses significantly outperformed sinusoidal ones: the velocity RSD was 32.2% vs. 52.8%, the Darcy force RSD (z) was 41.7% vs. 62.2%, and the radial surface tension RSD was 59.7% vs. 67.1%. This may be due to the cosinusoidal profile delivering peak power at the start of the pulse, followed by a gradual decline, allowing oscillations to settle before the next pulse. In contrast, sinusoidal pulses sustained excitation longer, promoting inter-pulse oscillations. However, the stabilizing advantage of the cosinusoidal profiles diminished at higher frequencies. By 400 Hz and 800 Hz, differences between the two shapes became negligible or even reversed slightly. For example, at 800 Hz and 4000 W, the radial surface tension RSD was 51.6% for cosinusoidal and 50.0% for sinusoidal pulses. Consequently, shape–frequency interaction was important in the optimization procedure. Additionally, radial fluctuations were consistently higher than axial ones under the same conditions, indicating that lateral oscillations (widening/narrowing) of the keyhole and melt pool were more pronounced than vertical (depth) fluctuations.

### 4.3. GRA and ANOVA

To evaluate the overall welding behavior across all eight response variables, the GRA was conducted on the 32 Taguchi-designed trials. Each response was first normalized according to its objective functions specified in Table 4. The GRA analysis and the corresponding ANOVA results are presented in this section.

#### 4.3.1. Response Table for GRG

The GRC for each response (Table 6) was averaged to obtain the GRG for each trial, serving as an overall performance index. Higher GRG values corresponded to deeper and wider welds with reduced fluctuations, instabilities, and thermal load. The GRG values ranged from approximately 0.44 (lowest quality) to 0.67 (highest quality), indicating significant variation in weld quality across different pulsing conditions.

The GRG-based performance ranking reveals clear trends related to pulse shape, frequency, and amplitude. As shown in Table 6, the highest GRG (0.666) was obtained for Test 25 (cosinusoidal, 400 Hz, 1000 W), followed by Test 17 (cosinusoidal, 100 Hz, 1000 W, GRG = 0.649) and Test 29 (cosinusoidal, 800 Hz, 1000 W, GRG = 0.626). Notably, six of the top eight tests used the lowest amplitude (1000 W), indicating that low power amplitude significantly improved the overall weld quality when all eight criteria were considered. Although lower amplitudes resulted in reduced penetration depth, this was offset by substantial improvements in process stability. Stability-related responses (e.g., velocity and force fluctuations) showed GRC values close to 1 (low variability and high stability) at low amplitudes, while high-amplitude conditions often had GRCs near 0.33 (less stability). Conversely, high amplitudes yielded higher GRCs for depth and width, but overall GRG values favored the stability benefits of lower power amplitude. Thus, GRA effectively balanced competing objectives, and the optimal performance occurred at the lowest tested amplitude. Pulse frequency also influenced weld quality. The average GRG was highest at 100 Hz (~0.557) and declined with increasing frequency. However, the top-ranked case occurred at 400 Hz, suggesting that an optimal combination of pulse shape and amplitude can compensate for the negative effects of higher frequency. This highlights the importance of interaction effects between parameters. Pulse shape had a smaller main effect compared to amplitude and frequency, but still played a role. On average, the cosinusoidal profile yielded a higher mean GRG (0.536) than the sinusoidal profile (0.504). This was especially evident at low amplitude, where early peak power in cosinusoidal pulses likely aided initial keyhole formation without destabilizing the melt pool. For example, at 100 Hz and 1000 W, the cosinusoidal case (Test 17, GRG = 0.649) significantly outperformed its sinusoidal counterpart (Test 1, GRG = 0.512), suggesting that an early power surge was beneficial at low frequency. Similar trends were observed at 400 Hz and 800 Hz. However, at higher amplitudes or when averaged across all frequencies, the shape advantage diminished. Thus, pulse shape alone had a modest effect (ranked third among the three factors), but its impact emerged in combination with frequency and amplitude settings. Overall, amplitude had the strongest influence on GRG, followed by frequency, and then shape. Lower amplitude and frequency, especially with a cosinusoidal waveform, provided the most favorable combination of weld quality and process stability. Conversely, the lowest-ranked tests often involved high frequencies and higher amplitudes with sinusoidal profiles.

#### 4.3.2. ANOVA Analysis of GRG and Key Factor Effects

To evaluate the statistical significance and influence of each control factor on overall welding performance, ANOVA was conducted on the GRG results. Table 7 summarizes the effects of the three main factors, including pulse shape (A), frequency (B), and amplitude (C), and their two-way interactions (A × B, A × C, B × C). The analysis revealed that all three main factors and the major interactions had a significant effect on the GRG (*p*-values < 0.01). The error term was very small (only ~1.4% of the total variation), indicating that the chosen factors and interactions explained ~98.6% of the variability in the multi-response performance. This confirms the effectiveness of the proposed optimization design in capturing the key influences on weld quality.

Among the main factors, laser power amplitude (Factor C) had the strongest influence, accounting for 51.9% of the total GRG variation. This quantitatively confirms its dominant role, as previously observed in the GRA trends. While higher amplitudes improved penetration, they also increased melt pool turbulence, reducing the overall GRG. In contrast, lower amplitudes enhanced stability and yielded a better balance between penetration and process control, supporting the recommendation to minimize amplitude within practical limits for improved weld quality. Pulse frequency was the third most influential factor (10.2% of GRG variation), followed by pulse shape (5.7%), which also showed statistically significant effects. Cosinusoidal waveforms generally outperformed sinusoidal ones, especially at low amplitudes and frequencies, due to their early energy delivery, which promoted more stable keyhole formation. Significant interaction effects were also observed: frequency–amplitude accounted for 23.8% of GRG variation, while shape–frequency and shape–amplitude interactions contributed approximately 3–4% each. These results highlight the importance of tuning parameter combinations rather than individual factors alone. For instance, high amplitude was more tolerable at low frequencies but led to substantial instability at high frequencies. Similarly, the benefits of cosinusoidal pulses were most pronounced under low-frequency, low-amplitude conditions, diminishing at higher frequencies.

## 5. Conclusions

This study introduced a Taguchi-based multi-response optimization approach using GRA to determine the optimal conditions for keyhole-mode laser spot welding of aluminum. The effects of a novel cosinusoidal laser power profile were evaluated alongside sinusoidal and continuous profiles. Welding performance was analyzed and optimized based on eight key response variables, including keyhole depth and width (larger-the-better), and the relative standard deviations of surface tension, Darcy damping force, and fluid velocity (smaller-the-better). The key findings are summarized as follows:

- Seven of the first ten best cases in terms of the overall welding performance belonged to the cosinusoidal pulse shape. A cosinusoidal pulse shape (test 25) with 1000 W amplitude and 400 Hz was found to be the best case, while a sinusoidal counterpart (test 15) with 800 Hz and 4000 W amplitude had the worst performance.
- The GRA results demonstrate that the best welding performances were achieved at a low amplitude of 1000 W (six among the top ten performances). However, increasing the amplitude to its maximum (4000 W) reached its best performance (fourth place among all) only when the frequency was set to its minimum (100 Hz).
- High-frequency and high-power amplitude pulses tend to destabilize the process and degrade the multi-objective outcome, whereas low-amplitude power, slow pulses foster stable keyhole dynamics and uniform heating, even if the penetration is lower.
- Selecting lower frequencies (100–400 Hz) is crucial for maximizing weld quality, offering adequate penetration with significantly improved process stability.
- Amplitude, frequency, and the two-way interaction between them contributed the most to the weld quality and system performance, with amplitude having the most contribution.

## Figures and Tables

**Table 1:** Thermophysical properties of 6061-T6 aluminum alloy [20,21].

Property	Symbol	Magnitude
Solidus temperature	$T_{s}$	873.13 (K)
Liquidus temperature	$T_{l}$	915.15 (K)
Vaporization temperature	$T_{V}$	2760 (K)
Thermal conductivity of solid	$k_{s}$	235 (W/m/K)
Thermal conductivity of liquid	$k_{l}$	90 (W/m/K)
Density of solid	$\rho_{s}$	2660 (kg/m^3^)
Density of liquid	$\rho_{l}$	2380 (kg/m^3^)
Latent heat of melting	$L_{m}$	3.87 × 10^5^ (J/kg)
Latent heat of vaporization	$L_{V}$	1.05 × 10^7^ (J/kg)
Specific heat capacity of solid	$C_{p,s}$	870 (J/kg/K)
Specific heat capacity of liquid	$C_{p,l}$	1170 (J/kg/K)
Convective heat transfer coefficient	h	20 (W/m^2^/K)
Coefficient of linear thermal expansion	β	2.8 × 10^−5^ (1/K)
Dynamic viscosity	μ	1.3 × 10^−3^ (Pa.s)
Coefficient of surface tension	σ	0.95 × (1 + 0.13 × (1 − T/T_m_))^1.67^ (N/m)
Temperature-dependent surface tension coefficient	∂σ/∂T	−0.15 × 10^−3^ (N/m/K)
Radiation emissivity	ξ	0.2

**Table 2:** Investigated factors and levels.

Symbol	Parameter	Level			
		1	2	3	4
A	Pulse Shape	Sinusoidal	Cosinusoidal	N/A	N/A
B	Frequency (Hz)	100	200	400	800
C	Amplitude (W)	1000	2000	3000	4000

**Table 3:** Chosen L32 mixed OA values.

Experiments	A	B	C
1	1	1	1
2	1	1	2
3	1	1	3
4	1	1	4
5	1	2	1
6	1	2	2
7	1	2	3
8	1	2	4
9	1	3	1
10	1	3	2
11	1	3	3
12	1	3	4
13	1	4	1
14	1	4	2
15	1	4	3
16	1	4	4
17	2	1	1
18	2	1	2
19	2	1	3
20	2	1	4
21	2	2	1
22	2	2	2
23	2	2	3
24	2	2	4
25	2	3	1
26	2	3	2
27	2	3	3
28	2	3	4
29	2	4	1
30	2	4	2
31	2	4	3
32	2	4	4

**Table 4:** Chosen L32 mixed orthogonal array values.

Parameter	Objective	Reason
Keyhole depth/width	Higher the better	Deeper welds
Mean temperature	Smaller the better	Less overall material heat exposure
RSD of Darcy damping force	Smaller the better	Reduced variability of parameter over time Fewer fluctuations; less instability
RSD of surface tension force		
RSD of velocity		

**Table 5:** The obtained test results for eight response variables under varying frequencies and amplitudes (RSD Sur_r,z_: RSD of surface tension in the radial or axial direction).

Test	Keyhole Depth (mm)	Keyhole Width (mm)	RSD Velocity (%)	Mean Temperature (K)	RSD Darcy_r_ (%)	RSD Darcy_z_ (%)	RSD Sur_r_ (%)	RSD Sur_z_ (%)
1	0.793	1.500	46.9	2682	62.4	56.8	42.4	23.1
2	0.930	1.610	53.2	2403	58.0	72.0	54.8	36.9
3	1.067	1.670	56.8	2112	74.4	62.6	62.0	39.8
4	1.218	1.728	52.8	1941	77.8	62.2	67.1	39.5
5	0.852	1.434	44.9	2684	60.3	52.5	35.3	22.3
6	0.951	1.516	48.8	2439	58.7	63.4	48.9	34.9
7	1.008	1.580	53.3	2152	71.5	58.0	54.4	38.9
8	1.116	1.616	47.9	1995	78.4	58.2	57.8	38.9
9	0.848	1.384	46.4	2692	58.5	42.8	27.0	23.0
10	0.880	1.446	54.4	2543	63.2	55.6	43.8	28.6
11	0.945	1.488	62.4	2298	62.9	57.3	50.9	36.7
12	0.997	1.508	59.8	2135	75.2	55.4	53.0	37.6
13	0.784	1.350	53.9	2698	56.9	39.8	23.6	27.4
14	0.777	1.396	58.2	2665	59.1	46.9	35.6	26.8
15	0.801	1.444	54.5	2531	67.4	59.2	46.5	28.7
16	0.886	1.502	62.0	2358	67.2	59.9	50.0	31.1
17	0.754	1.350	30.6	2695	53.3	45.7	28.2	23.7
18	0.776	1.436	29.5	2382	54.1	49.8	51.2	35.7
19	0.857	1.474	29.5	2114	73.4	43.9	56.5	34.5
20	0.984	1.514	32.2	1948	75.1	41.7	59.7	33.5
21	0.762	1.404	42.8	2697	55.0	40.5	29.9	25.0
22	0.784	1.492	44.6	2444	56.4	56.3	49.0	34.1
23	0.881	1.536	52.0	2162	73.5	49.8	56.2	36.5
24	1.062	1.576	54.1	1999	73.4	48.3	60.9	35.0
25	0.810	1.396	45.2	2697	53.4	39.6	26.7	21.8
26	0.903	1.442	53.0	2548	59.6	55.0	43.8	27.1
27	0.995	1.496	62.2	2293	69.4	57.4	52.8	35.7
28	1.076	1.528	64.6	2125	74.2	58.5	57.7	37.4
29	0.839	1.360	54.6	2689	51.2	41.2	25.0	25.8
30	0.863	1.380	58.7	2641	54.4	48.4	39.9	24.8
31	0.882	1.420	57.4	2505	63.1	61.8	49.8	27.8
32	0.931	1.466	62.4	2344	66.4	57.9	51.6	32.2

**Table 6:** GRC of the response variable results, their corresponding GRG for each test, and their ranked performance. (All GRC and GRG values are dimensionless quantities ranging from 0 to 1; RSD Sur_r,z_: RSD of surface tension in the radial or axial direction).

Test	GRC								GRG	Rank
	Keyhole Depth	Keyhole Width	RSD Velocity	Mean Temp	RSD Darcy_r_	RSD Darcy_z_	RSD Sur_r_	RSD Sur_z_		
1	0.35	0.45	0.50	0.34	0.55	0.48	0.54	0.88	0.511	15
2	0.45	0.62	0.43	0.45	0.67	0.33	0.41	0.37	0.465	24
3	0.61	0.77	0.39	0.69	0.37	0.41	0.36	0.33	0.491	17
4	1.0	1.00	0.43	1.00	0.34	0.42	0.33	0.34	0.607	5
5	0.39	0.39	0.53	0.34	0.60	0.56	0.65	0.95	0.551	11
6	0.46	0.47	0.48	0.43	0.64	0.41	0.46	0.41	0.470	21
7	0.52	0.56	0.42	0.64	0.40	0.47	0.41	0.34	0.472	20
8	0.69	0.63	0.49	0.88	0.33	0.47	0.39	0.34	0.527	14
9	0.39	0.35	0.51	0.34	0.65	0.86	0.86	0.88	0.605	7
10	0.41	0.40	0.41	0.39	0.53	0.50	0.52	0.57	0.466	23
11	0.46	0.44	0.35	0.51	0.54	0.48	0.44	0.38	0.449	27
12	0.51	0.46	0.37	0.66	0.36	0.51	0.43	0.36	0.457	26
13	0.35	0.33	0.42	0.33	0.71	0.99	1.00	0.62	0.592	8
14	0.34	0.36	0.38	0.34	0.63	0.69	0.64	0.64	0.505	16
15	0.36	0.40	0.41	0.39	0.46	0.45	0.49	0.57	0.440	32
16	0.41	0.46	0.35	0.48	0.46	0.44	0.45	0.49	0.443	31
17	0.33	0.33	0.94	0.33	0.87	0.73	0.83	0.83	0.649	2
18	0.34	0.39	1.00	0.46	0.83	0.61	0.44	0.39	0.559	10
19	0.39	0.43	1.00	0.69	0.38	0.79	0.4	0.41	0.561	9
20	0.45	0.47	0.87	0.98	0.36	0.89	0.38	0.43	0.609	4
21	0.34	0.37	0.57	0.33	0.78	0.95	0.77	0.74	0.607	6
22	0.35	0.44	0.54	0.43	0.72	0.49	0.46	0.42	0.482	19
23	0.41	0.50	0.44	0.63	0.38	0.61	0.40	0.38	0.468	22
24	0.6	0.55	0.42	0.87	0.38	0.65	0.37	0.40	0.530	13
25	0.36	0.36	0.53	0.33	0.86	1.00	0.88	1.00	0.666	1
26	0.42	0.40	0.43	0.38	0.62	0.51	0.52	0.63	0.489	18
27	0.51	0.45	0.35	0.52	0.43	0.48	0.43	0.39	0.444	29
28	0.62	0.49	0.33	0.67	0.37	0.46	0.39	0.37	0.462	25
29	0.38	0.34	0.41	0.34	1.00	0.91	0.94	0.69	0.626	3
30	0.40	0.35	0.38	0.35	0.81	0.65	0.57	0.75	0.532	12
31	0.41	0.38	0.39	0.40	0.53	0.42	0.45	0.60	0.448	28
32	0.45	0.42	0.35	0.48	0.47	0.47	0.44	0.46	0.443	30

**Table 7:** Results of ANOVA analysis (contribution was calculated as (Adj SS of Factor/total Adj SS) × 100).

Analysis of Variance						
Source	DOF	Adj SS	Adj MS	F-Value	*p*-Value	Contribution (%)
Amplitude	3	0.076427	0.025476	111.28	0	51.9
Pulse Shape	1	0.008454	0.008454	36.93	0	5.7
Frequency	3	0.015007	0.005002	21.85	0	10.2
Frequency × Amplitude	9	0.035032	0.003892	17	0	23.8
Pulse Shape × Amplitude	3	0.005416	0.001805	7.89	0.007	3.7
Pulse Shape × Frequency	3	0.004968	0.001656	7.23	0.009	3.4
Error	9	0.00206	0.000229			
Total	31	0.147366				

## Data Availability

The original contributions presented in the study are included in the article. Further inquiries can be directed to the corresponding author.

## Nomenclatures

$T_{m}$	Melting temperature; [K]
$T_{V}$	Vaporization temperature; [K]
$T_{s}$	Solidus temperature; [K]
T	Temperature; [K]
${dT}_{m}$	Smoothing interval of melting; [K]
${dT}_{V}$	Smoothing interval of vaporization; [K]
$k_{s}$	Thermal conductivity of solid; [W/m/K]
$k_{l}$	Thermal conductivity of liquid; [W/m/K]
$L_{m}$	Latent heat of fusion; [J/kg]
$L_{v}$	Latent heat of evaporation; [J/kg]
R	Universal gas constant; [J/mol/K]
${Cp}_{s}$	Specific heat of solid; [J/kg/K]
${Cp}_{l}$	Specific heat of liquid; [J/kg/K]
$\mu_{s}$	Dynamic viscosity of solid; [Pa.s]
$\mu_{l}$	Dynamic viscosity of liquid; [Pa.s]
d	Form factor for Gaussian distribution
C	Coefficient in Darcy’s law
b	Coefficient in Darcy’s law
$R_{eff}$	Effective radius of a laser beam; [m]
d	Dendrite dimension; [m]
M	Molecular mass of aluminum; [kg/mol]
h	Convective heat transfer coefficient; [W/m^2^/K]
f	Laser frequency; [Hz]
A	Laser power amplitude [W]
$\vec{g}$	Gravity; [m/s^2^]
p	Pressure; [atm]
$\vec{u}$	Velocity; [m/s]
t	Time; [s]
$F_{Darcy}$	Darcy damping force; [N/m^3^]
$F_{Buoyancy}$	Buoyancy force; [N/m^3^]
$V_{f,1}$	Volume fraction of fluid 1
$V_{f,2}$	Volume fraction of fluid 2
$D_{m}$	Gauss function around the melting temperature
$D_{V}$	Gauss function around the vaporization temperature
K	Constant representing the mushy zone morphology; [1/m^2^]
$P_{sat}$	Saturated vapor pressure; [atm]
$P_{atm}$	Atmospheric pressure; [atm]
$V_{L}$	Volume fraction of liquid
$V_{s}$	Volume fraction of solid
$\vec{n}$	Normal vector on the vapor/liquid interface
$\vec{k}$	Tangential vector on the vapor/liquid interface
$B_{t}$	Temporal laser distribution function used to apply pulses
n	Simulation steps
$x_{j}^{\left(i\right)}\left(t\right)$	Time-dependent value of the j-th response for the i-th test at time step t
${\bar{x}}_{j}^{\left(i\right)}$	Mean of $x_{j}^{\left(i\right)}\left(t\right)$ over all simulation steps sampled from t = 0 to t = 0.01 s
$X_{ij}$	Original values of the j-th response of the i-th test
${X_{ij}}^{*}$	Normalized values of the j-th response of the i-th test
$X_{j}$	Set of all values of the j-th response across all test cases ($X_{j}=\left\{X_{1j},\;X_{2j},\ldots X_{32j}\right\}$).
${∆}_{ij}$	Deviation of the normalized response from the ideal normalized response
${∆}_{min}$	Minimum possible deviation
${∆}_{max}$	Maximum possible deviation
r	Radial direction in the 2D axisymmetric design
z	Axial direction in the 2D axisymmetric design
Greek	
γ	Level-set parameter; [m/s]
∆	Deviation between two values
ε	Level-set parameter; [m]
δ	Delta function
ϕ	Level-set function (variable)
α	Absorptivity of aluminum on 1064 nm laser
ξ	Surface emissivity; distinguishing coefficient ranges from 0 to 1
$\beta_{L}$	Thermal expansion coefficient; [1/K]
$\beta_{R}$	Retro-diffusion coefficient
ρ	Density; [kg/m^3^]
μ	Dynamic viscosity; [Pa.s]
σ	Surface tension coefficient; [N/m]
Subscript	
L	Liquid
V	Vapor/vaporization
m	Melting; total number of responses in the GRA approach
Vol	Volume force
g	Gas
st	Surface tension
ls	Level set
Abbreviation	
ANOVA	Analysis of variance
GRA	Grey relational analysis
GRC	Grey relational coefficient
GRG	Grey relational grade
RSD	Relative standard deviation
SD	Standard deviation
LS	Level set
TEPT	Thermal-enthalpy porosity technique
MMT	Modified mixture theory
PW	Pulsed wave
CW	Continuous wave
PWM	Power wave modulation
HAZ	Heat affected zone
DOF	Degrees of freedom
OA	Orthogonal array
Sur	Surface tension
Adj	Adjusted
SS	Sum of squares
MS	Mean squares
CV	Cross-sectional view
TV	Top view
